# Dual effects of the Nrf2 inhibitor for inhibition of hepatitis C virus and hepatic cancer cells

**DOI:** 10.1186/s12885-018-4588-y

**Published:** 2018-06-25

**Authors:** Yuko Murakami, Kazuo Sugiyama, Hirotoshi Ebinuma, Nobuhiro Nakamoto, Keisuke Ojiro, Po-sung Chu, Nobuhito Taniki, Yoshimasa Saito, Toshiaki Teratani, Yuzo Koda, Takahiro Suzuki, Kyoko Saito, Masayoshi Fukasawa, Masanori Ikeda, Nobuyuki Kato, Takanori Kanai, Hidetsugu Saito

**Affiliations:** 1 Division of Pharmacotherapeutics, Faculty of Pharmacy, Keio University, Minato-ku, Tokyo, 105-8512 Japan; 2 Division of Gastroenterology and Hepatology, Department of Internal Medicine, Keio University, Shinanomachi 35, Shinjuku-ku, Tokyo, 160-8582 Japan; 3 Digestive Disease Center, International University of Health and Welfare, Mita Hospital, Tokyo, Japan; 4 Innovative Research Division, Mitsubishi Tanabe Pharma Corporation, Yokohama, Japan; 5 Department of Biochemistry and Cell Biology, National Institute of Infectious Disease, Tokyo, Japan; 6 Division of Persistent and Oncogenic Viruses, Center for Chronic Viral Diseases, Kagoshima University Graduate School of Medicine, Tokyo, Japan; 7 Department of Molecular Biology, Okayama University Graduate School of Medicine, Dentistry, and Pharmaceutical Sciences, Okayama, Japan

**Keywords:** Hepatitis C virus, Hepatocellular carcinoma, Nuclear factor E2-related factor 2, Chemotherapy, Brusatol, Sorafenib, Anticancer, Anti-HCV

## Abstract

**Background:**

We previously showed that knockdown of nuclear factor E2-related factor 2 (Nrf2) resulted in suppression of hepatitis C virus (HCV) infection. In this study, whether brusatol, an Nrf2 inhibitor, has dual anti-HCV and anticancer effects was explored.

**Methods:**

The anti-HCV effect of brusatol was investigated by analyzing HCV RNA and proteins in a hepatic cell line persistently-infected with HCV, HPI cells, and by analyzing HCV replication in a replicon-replicating hepatic cell line, OR6 cells. Then, dual anti-HCV and anticancer effects of brusatol and enhancement of the effects by the combination of brusatol with anticancer drugs including sorafenib, which has been reported to have the dual effects, were then investigated.

**Results:**

Brusatol suppressed the persistent HCV infection at both the RNA and protein levels in association with a reduction in Nrf2 protein in the HPI cells. Analysis of the OR6 cells treated with brusatol indicated that brusatol inhibited HCV persistence by inhibiting HCV replication. Combination of brusatol with an anticancer drug not only enhanced the anticancer effect but also, in the case of the combination with sorafenib, strongly suppressed HCV infection.

**Conclusions:**

Brusatol has dual anti-HCV and anticancer effects and can enhance the comparable effects of sorafenib. There is therefore the potential for combination therapy of brusatol and sorafenib for HCV-related hepatocellular carcinoma.

**Electronic supplementary material:**

The online version of this article (10.1186/s12885-018-4588-y) contains supplementary material, which is available to authorized users.

## Background

Chronic infection with hepatitis C virus (HCV) has been a worldwide health problem for decades, frequently leading to serious liver diseases such as liver cirrhosis and hepatocellular carcinoma (HCC) [1, 2]. For a long period, an interferon-based regimen has been the major therapy for HCV despite various adverse effects. Recently, several kinds of direct-acting antivirals (DAAs), which target proteins of the replication complex of HCV, including the nonstructural protein (NS)3, NS5A, and NS5B, have been developed, and combination regimens of such DAAs have achieved a sustained viral response more than 90% of the patients without using interferons [3]. It is known that reduction of persistent HCV infection reduces the incidence of HCC in HCV patients [4]. However, DAA treatments for HCV patients complicated with HCC are controversial because HCC as well as decompensated liver cirrhosis is a stronger prognostic factor than elimination of HCV for such patients [5]. We considered that one feasible resolution for this issue is the development of a drug that has dual effects, i.e., a drug that has both anti-HCV and anti-HCC effects.

Regarding agents with such dual effects, it has been demonstrated that the anti-tumor drug sorafenib, a kinase inhibitor that blocks the RAF kinase [6], also suppresses HCV replication, albeit in vitro [7–9]. Clinically, sorafenib has been approved and used for systemic anti-HCC therapy [10, 11]. However, sorafenib has not achieved a satisfactory cure of HCC [12]. Additionally, sorafenib did not affect the HCV RNA level during its clinical use in HCC patients with HCV [13]. Therefore, development of another agent with such dual effects is desirable for use as a monotherapy or as a combination therapy with existing anticancer drugs such as sorafenib.

Recently, we established a cell line persistently-infected with HCV, HPI cells, and showed that higher expression of nuclear factor E2-related factor 2 (Nrf2) contributes to persistent HCV infection, and that knockdown of Nrf2 suppresses its persistent infection [14]. Nrf2 is a transcriptional regulator of an array of genes including genes involved in the regulation of cell proliferation, redox homeostasis [15, 16] and cell metabolism such as glucose and glutamine metabolism [17]. Under normal conditions, Nrf2 is constantly degraded via ubiquitination by the association with Kelch-like ECH-associated protein 1 (Keap1) in the cytosol. Nrf2 is activated via dissociation with Keap1 by stress, such as reactive oxygen species. Nrf2 is also activated by phosphorylation independent of the Keap1 pathway in some tumors. Once it is activated in either way, Nrf2 or phosphorylated Nrf2 (p-Nrf2) is translocated into the nucleus and transactivates its target genes [18, 19].

Clinical studies have shown that Nrf2 is related to cell proliferation and invasion, as well as chemo-resistance of various human cancers [20–26], and that it is also involved in the progression and prognosis of HCC [20, 23, 24]. Indeed, somatic mutations of Nrf2 and Keap1 are detected in HCC [27], and a recent exome analysis showed that both Nrf2 and Keap1 are driver genes for carcinogenesis in HCC [28]. In addition, it has been reported that HCV activates Nrf2 [29], and that p62/Sqstm1 pathways were facilitated in HCV-positive HCC (C-HCC), leading to Nrf2-dependent metabolic reprogramming and promotion of HCC [30]. Based on these data, we expected that inhibition of Nrf2 could exert dual effects against HCV infection and proliferation of C-HCC.

Thus, to explore such dual effects by an Nrf2 inhibitor in vitro, we chose the quassinoid, brusatol, a compound derived from a natural product, which has been shown to inhibit the Nrf2 pathway and to reduce tumors in vivo and in vitro [31, 32]. Two cell lines, the HPI cell line and the OR6 cell line, were mainly used. The OR6 cell line is a full-length HCV replicon replicating cell line in which HCV replication can be easily determined by luciferase assay [33]. In these studies, it was demonstrated, for the first time, that brusatol has dual anti-HCV and anticancer effects.

## Methods

### Hepatocellular carcinoma tissue specimens

A tissue array slide of HCV-positive hepatocellular carcinoma tissue specimens including control non-hepatocellular-carcinoma specimens without either HCV or HBV infection (code LV8013) was purchased (US Biomax, Rockville, MD).

### Cell culture

The HPI cell line, which was established in our previous study [13], and the hepatoma cell lines Huh6 [34], Huh7.5 [35], and HepG2 (ATCC #HB-8065), were cultured in high-glucose DMEM (Life Technology, Carlsbad, CA) supplemented with 10% fetal calf serum. The OR6 cell line was cultured in the same medium with addition of 0.3 mg/ml geneticin (Life Technologies).

### Knockdown experiment

Knockdown for Nrf2 expression in cultured cells was performed by transfection of siRNA against Nrf2, NEF2L2-HSS107128 (Life Technologies) and control RNA, stealth RNAi negative control medium GC duplex (Life Technologies). The transfection was done with Lipofectamine RNSiMAX transfection reagent (Life Technologies) according to the manufacturer’s protocol.

### Immunohistochemical staining

A tissue array slide was deparaffinized and hydrated with xylene and a graded alcohol series. After antigen activation in 10 mM citrate at 120 °C for 10 min, non-specific binding was blocked with 5% bovine serum albumin and the slide was incubated overnight with a primary antibody at 4 °C. Subsequently, the slide was incubated with a secondary antibody for 1 h, and the signal was developed by staining with 3,3′-diaminobenzidine using the Vectastain Elite ABC Kit® (Vector Laboratories, Burlingame, CA) according to the manufacturer’s protocol. Positivity of p-Nrf2 was given an immunoreactivity score, which was determined by amplification of the intensity of nuclear staining score (0, negative; 1, weakly positive; 2, moderately to strongly positive) and the ratio of stained nuclei (0, no nuclei; 1, 1 to 50%; 2, more than 50%). The immunoreactivity scores were generated by a pathologist. Statistical analysis was done with the Mann-Whitney test using Graphpad (Prism, La Jolla, CA).

### Immunofluorescence staining

Cultured cells were seeded on a chamber slide 24 h prior to the administration of a reagent or 48 h prior to immunofluorescence staining without administration of a reagent. For immunofluorescence staining, the cells were fixed with 4% paraformaldehyde, permeabilized with 0.05% Triton X-100 solution and blocked with 5% bovine serum albumin. Subsequently they were incubated with primary antibodies (mixed) and then with secondary antibodies (mixed) against the respective primary antibody. Finally, the cells were mounted with Vectashield containing 4′,6-diamidino-2-phenylindole (Vector Laboratories). Immunofluorescence was detected and processed by using a fluorescence microscope, EVOS AMF-4302 (Thermo Fisher Scientific, Waltham, MA), and Photoshop CS (Adobe Inc., San Jose, CA). Subcellular localization, nuclear or cytosol, of the protein was determined by merging images of the protein and DAPI.

### Immunoblot analysis

For immunoblot analysis, cultured cells were harvested in RIPA buffer (Thermo Fisher Scientific, Waltham, MA). After the addition of an equal volume of 2X Laemmli sample buffer (Bio-Rad, Hercules, CA) containing 5% β-mercaptoethanol, the cell lysates were heat-denatured at 95 °C for 5 min and then sonicated for 10 min. The protein concentration of the sample was determined using the Pierce 660-nm Protein Assay kit (Thermo Fisher Scientific) according to the manufacturer’s protocol, and equal amounts (protein content) of samples were subjected to SDS-PAGE (Bio-Rad). Proteins in the gels were transferred to the PVDF membrane, Immobilon (Merck Millipore, Darmstadt, Germany), blocked with 5% milk powder, and incubated with a primary antibody at the concentration recommended by the manufacturer at room temperature for 1 h or at 4 °C overnight. Then, after incubation with horseradish peroxidase-conjugated secondary antibody (GE Healthcare, Little Chalfont, UK) for 1 h, the protein signals were detected by using ECL Prime (GE Healthcare). Relative intensity of the immunoblot band for the proteins to that of b-actin was calculated with the image analyzer at each time point and concentration. It was calculated with the values for no drug at 0 h as 1.00.

### Primary and secondary antibodies for immunohistochemical staining, immunofluorescence staining and immunoblot analysis

Primary antibodies used were against HCV core (Institute of Immunology, Tokyo, Japan), HCV NS5A (Virogen, Watertown, MA), β-Actin (Abcam, Cambridge, UK), Nrf2 (Santa Cruz, Dallas, TX), and p-Nrf2, phospho-serine 40, (Abcam). For immunoblot analysis, HRP-labeled secondary antibodies against mouse IgG, rabbit IgG, and goat IgG (GE Healthcare) were used depending on the primary antibodies used for immunoblotting. For immunofluorescence staining, Alexa-fluor-488-labeled goat anti-mouse and Alexa-fluor-568-labeled goat anti-rabbit (Life Technologies) secondary antibodies were used.

### Quantitative reverse transcription polymerase chain reaction (qRT-PCR)

qRT-PCR assays were performed with the Thermal Cycler Dice TP800 (Takara, Shiga, Japan) to measure RNA of 2 regions of the HCV genome: the 5′ untranslated region (5’UTR) and the NS5A region. Primer sets (forward / reverse) used were: 5’-AAGCGTCTAGCCATGGCGTTAGTA / 5’-GGCAGTACCACAAGGCCTTTCG, 5’-CCGCGACGTGTGGGACTGGGTTTGCAC / 5’-CTCCGAGGCCGCCACCCTCCAGATGGC and 5’-GCACCGTCAAGGCTGAGAAC / 5’-TGGTGAAGACGCCAGTGGA for the 5’UTR, the NS5A region and control GAPDH, respectively. The reverse transcription reaction was done at 37 °C for 15 m, and then at 85 °C for 5 s with the Primescript RT Master Mix (Takara). The polymerase chain reaction was done first at 95 °C for 30 s and then with 40 cycles of 95 °C for 5 s and 60 °C for 30 s, followed by 95 °C for 15 s, 60 °C for 30 s and 95 °C for 15 s, using the KAPA SYBR® FAST qPCR Kit (Kapa Biosystems, Boston, MA). Relative quantification was performed using the 2^-ΔΔCT^ method, and each value was normalized by the value of GAPDH. PCR amplifications were performed in triplicate, and statistical analysis was performed by using Student’s *t*-test.

### Luciferase assay

For luciferase assays, OR6 cells were seeded on a 24 well culture plate. The assay was performed in triplicate using a Renilla Luciferase Assay System (Promega, Madison, WI) according to the manufacturer’s protocol. Luminescent signals were measured with a spectrometer (Promega).

### Transcriptome analysis

At 24 h after plating the HPI cells onto a 10 cm-diameter dish, 160 nM brusatol or 25 nM siRNA against Nrf2 together with control (DMSO and scrambled RNA, respectively) were added to the culture medium. The concentration of brusatol and siRNA corresponded to that which provides twice the 50% growth inhibition (GI50) and 14% inhibition of Nrf2 expression (based on the previous study), respectively. Forty-eight hours later, total RNA was prepared from the cultured cells using the RNeasy extraction kit (Qiagen, Germantown, MA). For transcriptome analysis, cDNA microarray analysis of the extracted RNA was performed using Human Oligo Chip 25 K (Toray, Tokyo, Japan) and 3D-Gene scanner 3000 (Toray). Microarray data were deposited in Gene Expression Omnibus (https://www.ncbi.nlm.nih.gov/geo/).

### Cell viability assay

Cell viability was determined using the MTS assay kit, Celltiter 96® Aqueous One Solution Cell Proliferation Assay (Promega) according to the attached protocol. The absorbance of each well was measured with the microplate reader Model 680 (Bio-Rad). At 48 h after the administration of the drug, a curve was created by plotting the logarithm of the concentration of the drug on the X-axis and the percentage of cell growth, determined with the MTS assay, to that of no drug on the Y-axis. Then, the concentration of the drug corresponding to 50% growth inhibition (GI50) was estimated by using Graphpad (Prism). As for viable cell counting with trypan blue, the HPI cells were seeded onto a 24-well plate. At indicated time points, the cells were washed, treated with trypsin-EDTA solution and stained with 0.2% trypan blue, and then non-stained cells were counted. Measurements for MTT assay and the trypan blue method were done in triplicate at each time point, and statistical analysis was performed by using Student’s *t*-test.

## Results

### Expression of p-Nrf2 in C-HCC and cultured hepatoma cell lines

First, we explored the expression status of the active form of Nrf2, p-Nrf2, was explored in C-HCC clinical specimens using a tissue array slide and cultured hepatoma cell lines including a HCV-positive cell line (HPI cell line) and HCV-negative cell lines (Huh7.5, Huh6 and HepG2 cell lines).

**Fig. 1:**
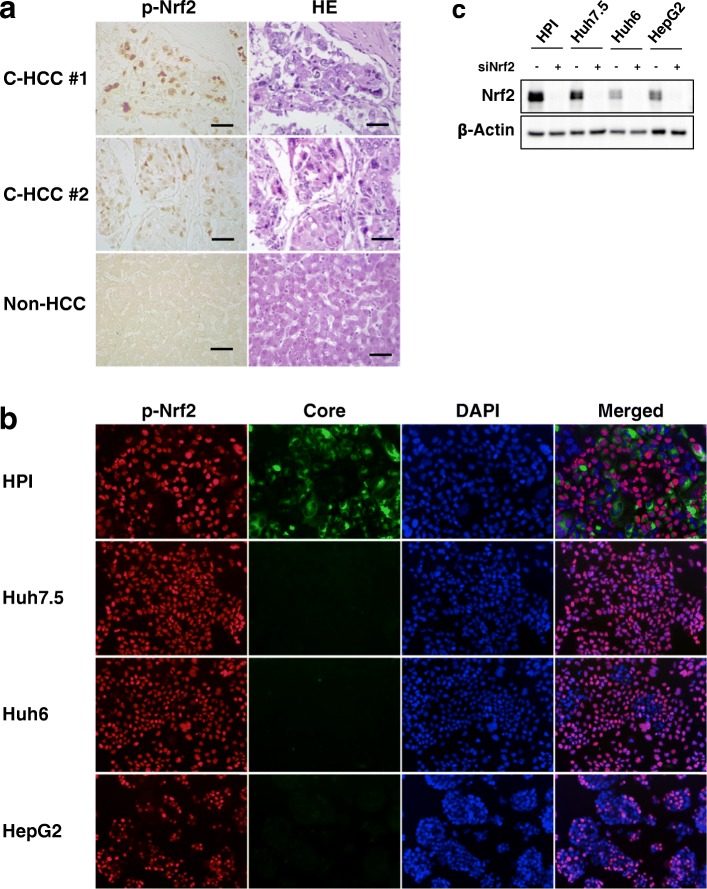
Expression of p-Nrf2 in C-HCC samples and in cultured hepatoma cell lines. **a** Immunohistochemical analysis of the expression of p-Nrf2, and hematoxylin-eosin (HE) staining in C-HCC specimens (#1 and #2) and a non-HCC specimen without either HCV or HBV infection. Bars, 50 μm. **b** Immunofluorescent analysis of the p-Nrf2 and the HCV core protein with nuclear staining (DAPI), and their merged images in the cell lines. **c** Immunoblot analysis of the Nrf2 protein in the cultured hepatoma cell lines after knockdown with siRNA against Nrf2 or control RNA. Beta-actin was used for validation of sample loading

Figure 1a shows representative features of p-Nrf2 expression in C-HCC clinical specimens as determined by immunohistochemistry. In this analysis, p-Nrf2 was expressed exclusively in the nuclei of 45% (9/20) of the C-HCC specimens on the array examined, whereas only 5% (1/20) of the non-HCC specimens without either HCV or HBV infection were positive for p-Nrf2. Positivity of p-Nrf2 as represented by an immunoreactivity score was significantly greater in C-HCC specimens (0, 55%; 1, 10%; 2, 25%; 10%; 4, 10%) than in non-HCC specimens (0, 95%; 1, 5%) (*p* = 0.003). As shown in the immunofluorescence analysis (Fig. 1b), p-Nrf2 was expressed exclusively in the nuclei of the HPI cells, in which HCV core protein was also positively stained in cytosol. p-Nrf2 was also exclusively expressed in the nuclei of the HCV-negative hepatoma cell lines, although relatively few of the HepG2 cells were p-Nrf2-positive. Expression of Nrf2 itself was not clearly recognized by immunofluorescent analysis owing to non-specific signals in the cytosol. Instead, we performed immunoblot analysis of Nrf2 after knockdown with siRNA against Nrf2 was performed, and it was confirmed that Nrf2 expression in the hepatoma cell lines was suppressed with siRNA against Nrf2 (Fig. 1c).

These results confirmed that p-Nrf2 was expressed in a considerable number of C-HCC clinical specimens as well as in the hepatoma cell lines including the HCV-positive hepatoma cell line, supporting the concept that p-Nrf2 plays an important role in the pathogenesis of C-HCC.

### Brusatol reduced the HCV RNA level in the HPI cells

**Fig. 2:**
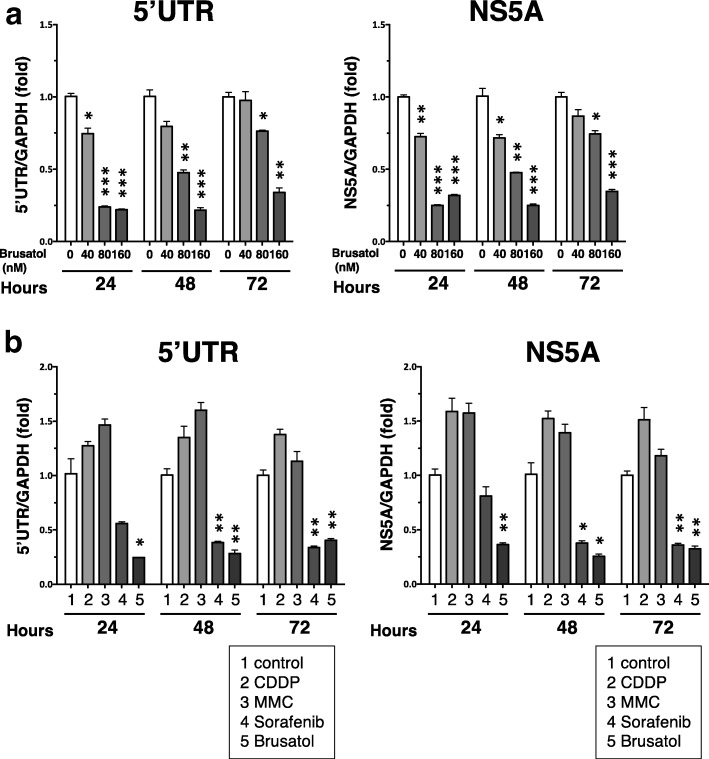
Effect of brusatol and anticancer drugs on the HCV RNA level. **a-b** qRT-PCRs for HCV RNA in the HPI cells. The value for HCV RNA was normalized by the value for GAPDH RNA and is presented as fold change to that of control. Statistical significance *: *p* < 0.01, **: *p* < 0.001, ***: *p* < 0.0001 versus 0 nM or control. **a** qRT-PCRs after the administration of brusatol. **b** qRT-PCRs after the administration of agents at a concentration for the GI50 (80 nM, 4.3 μg/ml, 2.0 μg/ml and 8.0 μM for brusatol, CDDP, MMC and sorafenib, respectively)

To determine if brusatol affects the persistence of HCV infection, the effect of brusatol administration on the RNA levels in the 5’UTR and the NS5A region of HCV in the HPI cells was analyzed by qRT-PCR. Brusatol reduced HCV RNA levels in a dose-dependent manner from 24 to 72 h after its administration (Fig. 2a). This effect was diminished at 48 h and 72 h compared to 24 h after the administration of brusatol, possibly because of recovery of RNA replication at the later time, especially in a lower concentration of brusatol.

To further explore this effect, this effect of brusatol was compared with that of anticancer drugs such as cis-diamminedichloro-platinum (CDDP), mitomycin C (MMC), and sorafenib. In order to adjust the effects on cell toxicity, they were administered at a concentration corresponding to GI50 (determined in Fig. 6a to d): 80 nM, 4.3 μg/ml, 2.0 μg/ml and 8.0 μM, for brusatol, CDDP, MMC and sorafenib, respectively. The extent of HCV RNA reduction by brusatol was comparable to that by sorafenib whereas CDDP and MMC did not induce a reduction in HCV RNA (Fig. 2b).

These results indicated that the potency of brusatol for suppression of the persistence of HCV infection at the RNA level was similar to that of sorafenib.

### Brusatol reduced the level of the Nrf2 protein and the HCV proteins in the HPI cells

**Fig. 3:**
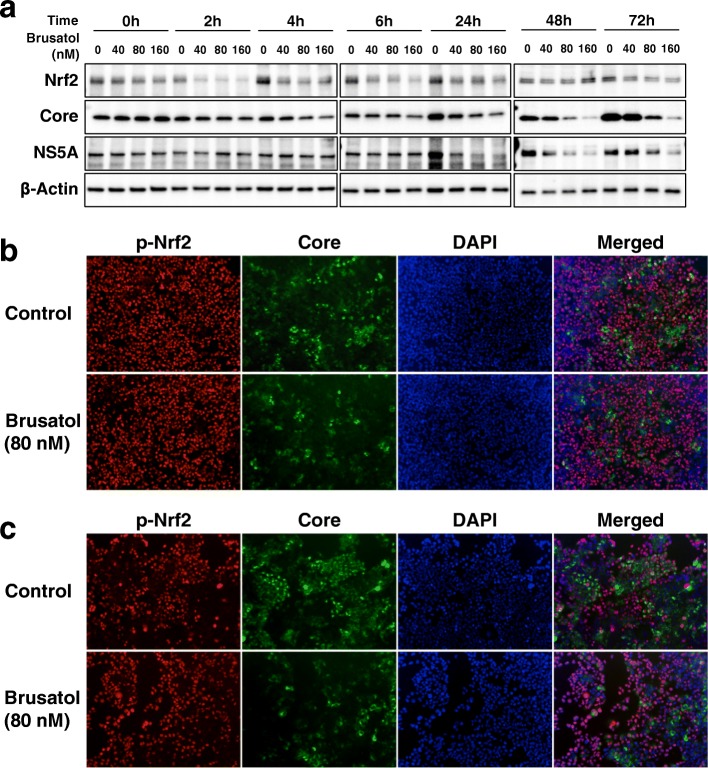
Effect of brusatol on expression and subcellular distribution of Nrf2 and HCV proteins. **a** Immunoblot analysis of Nrf2 and HCV proteins in HPI cells after administration of brusatol. Beta-actin was used for validation of sample loading. **b-c** Immunofluorescent staining for p-Nrf2 and the HCV core protein in the HPI cells with nuclear staining (DAPI) at 6 (**b**) and 48 (**c**) h after the administration of brusatol

To investigate the inhibitory effect of brusatol on HCV infection and Nrf2 at the protein level, the Nrf2 protein and the HCV proteins in the HPI cells were analyzed after the administration of brusatol using immunoblot analysis (Fig. 3a and Additional file 1: Table S1). In the absence of brusatol, the level of the Nrf2 protein increased gradually over the first 24 h, possibly reflecting robust cell proliferation early after cell seeding prior to contact inhibition. However, the increase in the Nrf2 protein during this period was suppressed by brusatol. As to the HCV proteins, the core and NS5A proteins were also suppressed by brusatol in a dose-dependent manner, especially from 24 h to 72 h after its administration, whereas in the absence of brusatol, their levels increased markedly for up to 72 h after the administration of DMSO (control).

**Fig. 4:**
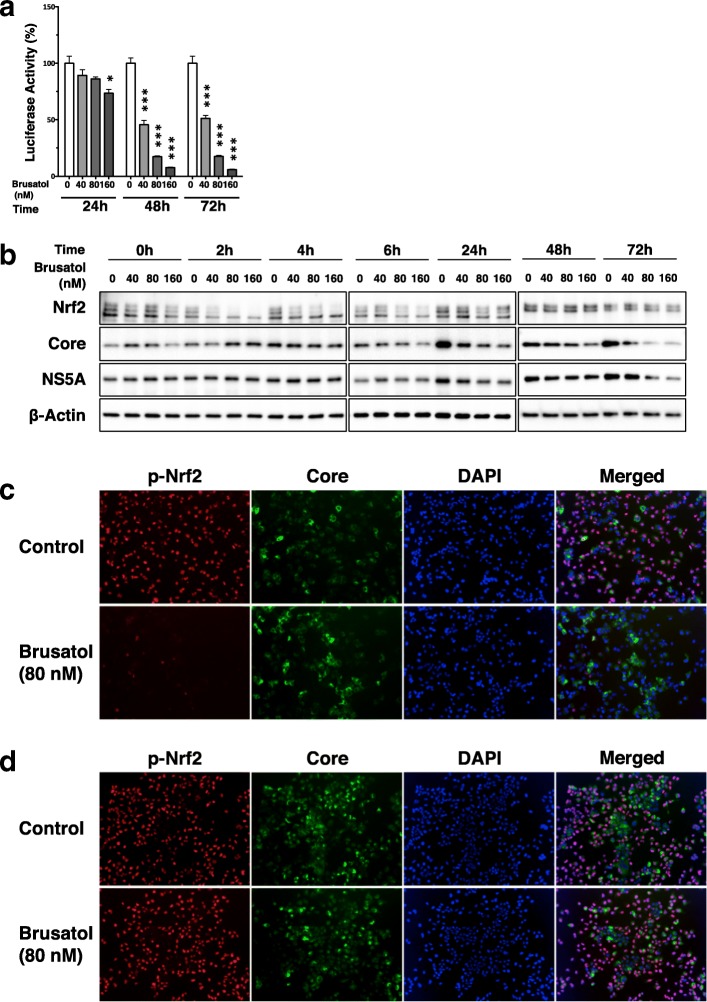
Effect of brusatol on replication of the HCV replicon. **a** Luciferase activity of OR6 cells after administration of brusatol. Statistical significance *: p < 0.01, **: p < 0.001, ***: p < 0.0001 versus 0 nM. **b** Immunoblot analysis of Nrf2 and HCV proteins in OR6 cells after administration of brusatol. Beta-actin was used for validation of sample loading. **c-d** Immunofluorescent staining of p-Nrf2 and the HCV core protein in OR6 cells with nuclear staining (DAPI) at 6 (**c**) and 48 (**d**) h after the administration of brusatol

Next, to explore the subcellular expression status of p-Nrf2 and the HCV proteins in the HPI cells after the administration of brusatol, immunofluorescent staining of these proteins was performed at 6 and 48 h after its administration (Fig. 3b and c, respectively). In accordance with the immunoblotting analysis, the cytosolic expression of the HCV core protein was suppressed at 48 h after brusatol administration albeit with a lower level of suppression at 6 h. However, the nuclear expression of p-Nrf2 with brusatol administration did not differ much from that of control at either time point. In contrast, pNrf2 was markedly reduced on immunofluorescence in OR6 cells after the administration of brusatol (Fig. 4c). This difference could be attributed to the characteristics of the both cells; while they were originated from Huh7.5 cells, OR6 cells and HPI cells were established after a few months and around 2 years, respectively. We speculated that the total amount of the Nrf2, as shown in Fig. 3a, is more crucial than the nuclear expression of p-Nrf2 in the HPI cells, and further study will be needed to explain this phenomenon.

These combined experiments, we verified that brusatol suppressed the persistence of HCV infection at the protein level, as well as at the RNA level, in association with reducing the Nrf2 protein level.

### Brusatol inhibited replication of HCV replicon

The infection cycle of HCV consists of multi-steps, such as viral entry, uncoating, translation, and replication and production of virus particles. Of these steps, we focused on the replication step as a candidate of a target of brusatol to suppress persistent infection of HCV, since the replication step is the most crucial step for persistence of HCV infection.

To evaluate the effect of brusatol on HCV replication, luciferase activity of the OR6 cells was measured after the administration of brusatol. Brusatol dramatically reduced luciferase activity in a dose-dependent manner from 48 h to 72 h after its administration (Fig. 4a). However, early after brusatol administration (at 43 h), suppression of HCV replication in OR6 cells was not as great as suppression of HCV RNA levels in the HPI cells (Fig. 2a). It is likely that this difference is related to the delay in brusatol-induced change in luciferase protein compare to brusatol-induced primary change in HCV RNA.

To explore the effect of brusatol on HCV replication at the HCV protein level in the OR6 cells, the expressions of the HCV proteins and the Nrf2 protein were analyzed by immunoblot analysis after the administration of brusatol using immunoblot analysis (Fig. 4b and Additional file 3: Table S2). While the Nrf2 protein level increased gradually over 24 h in the absence of brusatol, the Nrf2 protein level was suppressed by brusatol, although the suppression level was not as intense as was observed in the HPI cells. As to the HCV proteins, the core and NS5A proteins were markedly suppressed by the administration of brusatol in a dose-dependent manner from 24 h to 72 h, whereas the core and NS5A proteins increased in the absence of brusatol.

Next, to explore the subcellular expression status of p-Nrf2 and the HCV proteins in the OR6 cells after the administration of brusatol, immunofluorescent staining of these proteins was performed at 6 h and 48 h after its administration (Fig. 4c and d). Nuclear expression of p-Nrf2 was remarkably suppressed at 6 h after brusatol administration, but it had almost recovered by 48 h. On the other hand, cytosolic expression of the core protein was suppressed at 48 h, in accordance with the result of the immunoblot analysis.

These results suggest that suppression of persistent HCV infection by brusatol was due to its inhibition of HCV replication, although there remains a possibility that other steps of the infection cycle might also be involved.

### Comparison of the transcriptome of the HPI cells treated with brusatol and that of the cells treated with siRNA against Nrf2

**Fig. 5:**
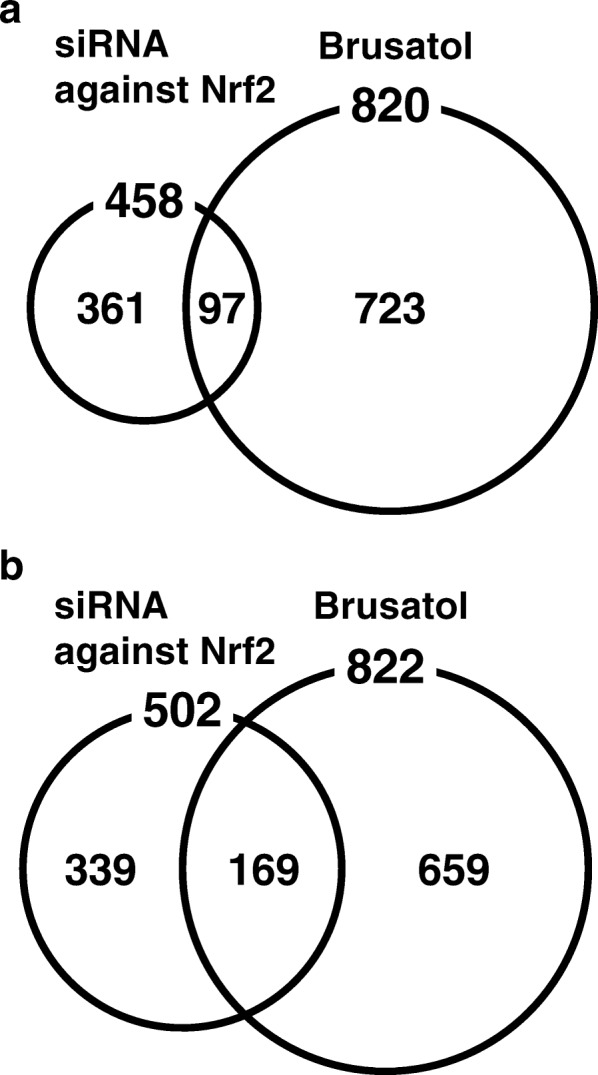
Venn-diagram of genes down-regulated and up-regulated by brusatol or by the siRNA against Nrf2. **a-b** Venn-diagrams of affected genes based on the transcriptome of the HPI cells treated with brusatol or with siRNA against Nrf2. The microarray data were deposited in Gene Expression Omnibus (accession numbers: GSE52321 and GSE98920). **a** The numbers of genes down-regulated (< 0.5-fold vs. control) by the siRNA or by brusatol are shown in the circles. The number of genes commonly down-regulated is shown in the overlapped region of the circles. The number of genes down-regulated exclusively by each agent is shown in the non-overlapped region; **b** The numbers of genes up-regulated (> 0.5-fold vs. control) by the siRNA or by brusatol are shown in the circles. The number of genes commonly up-regulated is shown in the overlapped region of the circles. The number of genes up-regulated exclusively by each reagent is shown in the non-overlapped region

We predicted that brusatol could affect expression of a wider range of genes than siRNA against Nrf2, which also suppresses persistent HCV infection in the HPI cells [32], because siRNA more specifically targets gene expression in general. To clarify this difference, the transcriptomes of the HPI cells treated with brusatol and that of the cells treated with siRNA against Nrf2 were compared. This analysis showed that 97 genes were commonly down-regulated by the two agents. The total number of genes down-regulated (less than 0.5-fold vs. control) by brusatol was greater than that of genes down-regulated by the siRNA against Nrf2 (820 vs. 458) (Fig. 5a). On the other hand, 169 genes were commonly up-regulated (more than 2-fold vs. control) by the two reagents. The total number of genes up-regulated by brusatol was greater than that of genes down-regulated by the siRNA against Nrf2 (822 vs. 502) (Fig. 5b).

The categories of gene function of the commonly affected genes are shown in Table 1. Notably, 33 of the 97 commonly down-regulated genes belonged to categories related to metabolisms including lipid metabolism (10 genes), cholesterol metabolism (6 genes), glutamine/glutamate metabolism (5 genes) and other types of metabolism (12 genes), while 54 of the 163 commonly up-regulated genes belonged to categories including transcription (30 genes), signal transduction (10 genes) and cell proliferation/growth (14 genes).

**Table 1:** Categoly of genes down-regulated and up-regulated in the HPI cell commonly by the treatment with brusatol and by the treatment with siRNA for Nrf2

Category of gene function	Corresponding GO number^a^	Number of genes commonly down-regulated (less than 0.5 fold)	Number of genes commonly up-regulated (more than 2 fold)
Lipid metabolism	GO:0006629, GO:0016042, GO:0030497	10	2
Cholesterol metabolism	GO:0006695, GO:0008203, GO:0016125	6	1
Glutamine/glutamate metabolism	GO:0006536, GO:0006542, GO:0006749	5	0
Other metabolisms	GO:0008152, GO:0005975	12	4
Oxidation reduction	GO:0055114, GO:0045454, GO:0006979	11	6
Inflammatory/immune response	GO:0006954, GO:0006955	3	4
Transcription	GO:0006810, GO:0015031	5	30
Signal transduction	GO:0007165,	6	10
Translation	GO:0006412	4	2
Protein/amino acids modification	GO:0006468, GO:0006470, GO:0006486, GO:0006493, GO:0006464	6	11
Transport	GO:0006810, GO:0015031	7	4
Biologiacal process	GO:0008150	2	5
Cell proliferation/growth	GO:0008285, GO:0008283, O:0007049, GO:0001558	5	14
Apoptosis	GO:0006917, GO:0042981, GO:0006917, GO:0043065	3	7
Cell adhesion	GO:0007155	3	6
Multicellular organismal development	GO:0007275	3	9
Other functions		12	25
Unknouwn functions		21	54
Total^b^		97	163

^a^gene ontology (GO) number based on Gene Ontology Consortium (http://www.geneontology.org/) ^b^some genes were overlapped as to category

These transcriptome analyses showed that brusatol affected a wider range of gene expression than siRNA against Nrf2, and that a considerable number of genes were commonly affected by the two agents especially in categories related to metabolisms, of which cholesterol metabolism is known to be of great importance for the infection cycle of HCV.

### Inhibition of the proliferation of the HPI cells by brusatol and anticancer drugs

**Fig. 6:**
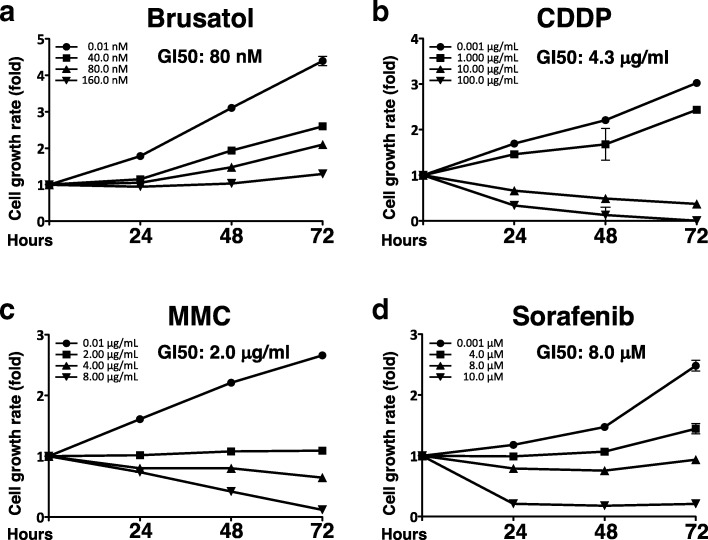
Effect of brusatol and anticancer drugs on the proliferation of the HPI cells. **a-d** Effect of brusatol or the anticancer drugs on the growth of HPI cells. Cell viability after the administration of brusatol (**a**), CDDP (**b**), MMC (**c**), or sorafenib (**d**) was determined with the MTS assay. The cell growth rate is presented as fold change relative to that of 0 h. The concentration of GI50 for each reagent was calculated using the data at 48 h

To confirm the inhibitory effect of brusatol on the proliferation of the HPI cells and to determine the concentration of brutasol required for 50% growth inhibition (GI50), the viability of the HPI cells after administration of brusatol (Fig. 6a) and, for comparison, after administration of anticancer drugs such as CDDP, MMC and sorafenib (Fig. 6b-d, respectively) was measured. Brusatol time-dependently and dose-dependently reduced cell viability, and the concentration for GI50 was calculated as 80 nM, 4.3 μg/ml, 2.0 μg/ml, and 8.0 μM for brusatol, CDDP, MMC and sorafenib, respectively. With trypan blue staining as well, brusatol time-dependently and dose-dependently reduced cell viability (Additional file 2: Figure S1), and the concentration for GI50 at 48 h was calculated as 91 nM, which almost corresponded to the value with the MTS assay (80 nM). However, the inhibition of cell proliferation by brusatol was not as potent as that of anticancer drugs even with a higher brusatol concentration (320 nM, data not shown), possibly due to the difference in the mechanism of inhibition of proliferation between brusatol and the anticancer drugs. The inhibition of cell proliferation of the other cell lines including Huh7.5, Huh6 and HepG2 cells by brusatol was confirmed (data not shown).

These results showed that brusatol has anticancer effect against hepatoma cell lines, although the effect was not as potent as the other anticancer drugs.

### Combination of brusatol and sorafenib simultaneously enhanced anticancer and anti-HCV effects

It is of clinical use to combine anticancer drugs with different mechanisms of action in order to enhance the total anticancer effect and to reduce the dosage of individual drugs to decrease adverse effects. Therefore, the effects of a combination of brusatol with an anticancer drug on both cell proliferation and HCV infection were investigated.

**Fig. 7:**
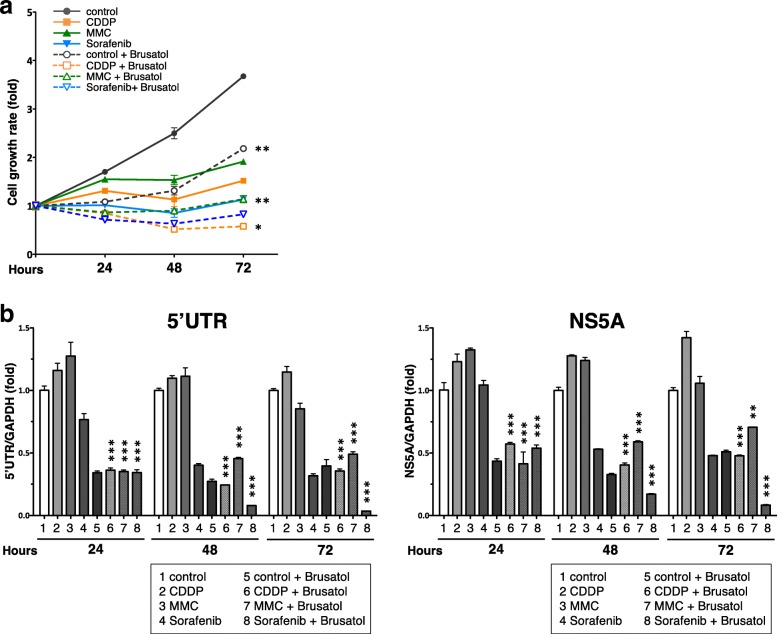
Effect of the combination of brusatol with anticancer drugs on cell proliferation and HCV infection. **a** Growth of the HPI cells after the administration of brusatol, the anticancer drugs or their combination (at a concentration of GI50). Cell viability was determined with the MTS assay. The cell growth rate is presented as fold change relative to that of 0 h. **b** qRT-PCR for the HCV RNA after the administration of brusatol, the anticancer drugs, or the combination of brusatol with the anticancer drugs (at a concentration of GI50). The value for the HCV RNA was normalized by the value for GAPDH RNA and is presented as fold change relative to the value in the absence of reagent. Statistical significance *: p < 0.01, **: p < 0.001, ***: p < 0.0001 versus the single administration of the corresponding anticancer drug

Proliferation of the HPI cells was inhibited by the combination of an anticancer drug with brusatol more effectively than by a single administration of the anticancer drug (Fig. 7a). Moreover, the combination of brusatol with an anticancer drug reduced the HCV RNA level in the HPI cells to the same extent as that of a single administration of brusatol at 24 h after their administration (Fig. 7b). However, at later time points, from 48 h and 72 h after the drug administration, only the combination of brusatol and sorafenib dramatically reduced the HCV RNA level, whereas the combination of brusatol with the anticancer drugs did not further reduce the HCV RNA level.

These data showed that the combination of brusatol with an anticancer drug enhanced the anticancer effect of the anticancer drugs. Most importantly, the combination of brusatol and sorafenib dramatically suppressed HCV infection in addition to enhancing the anticancer effect and, at least based on the value at 72 h (lanes 4, 5 and 8 in Fig. 7b), this effect of the combination could be synergistic on HCV suppression.

## Discussion

The present study showed that 35% (7/20) of C-HCC samples were positive for p-Nrf2. Although this percentage was less than the 55% (11/20) in HCC with HBV infection (data not shown), activation of Nrf2 is attributed to the pathogenesis of C-HCC. For clinical application of brusatol, however, the relationship between Nrf2 or p-Nrf2 expression status and clinicopathological features of HCC such as stage, histology, susceptibility and prognosis must be clarified. Since the results in the present study were obtained using only tissue array analysis, we are planning to conduct a cohort study of Nrf2 or p-Nrf2 expression in C-HCC.

We hypothesized that inhibition of Nrf2 could inhibit both HCV infection and proliferation of hepatoma cells based on our previous study and on previous reports that are described in the introduction. To date, a small number of Nrf2 inhibitors has been described including brusatol [31, 32], retinoic acid receptor α agonists [36], leutolin [37], and trigonelline [38]. Of them, we preliminary explored the anti-HCV effect via Nrf2 inhibition by using brusatol and all-trans retinoic acid (ATRA), which is known to have an anti-HCC effect [39]. We chose brusatol for the present study because only brusatol showed an anti-HCV effect but ATRA did not, although reason for this difference was not unclear.

Brusatol has been shown to have an anti-proliferation effect on cancer cells including chemoresistant cells. Although the precise mechanism by which brusatol inhibits Nrf2 is not fully understood, it was shown that brusatol and related compounds inhibits protein synthesis [40]. Furthermore, brusatol selectively inhibits the Nrf2 pathway, and the reduction of Nrf2 is through enhancement of ubiquitination and degradation of Nrf2 [31]. Therefore, the alteration of mRNA expression observed in the present study could be a secondary phenomenon after reduction of Nrf2 protein caused by brusatol. A recent study demonstrated that brusatol reduced the Nrf2 protein level in a post-translational manner, since this reduction appeared very early (from 30 min to 12 h) after its administration, with maximal inhibition at around 2 h [32]. The present study using the HPI cells similarly showed that reduction of Nrf2 was maximal at 2 h after the administration of brusatol supporting the post-translational mechanism for the reduction in the Nrf2 protein level by brusatol. However, in the present study, the effect of brusatol continued for a relatively longer time (more than 24 h) than in the previous study, possibly because of differences in the experimental conditions including the cell line that was used.

We considered that the suppression of HCV by brusatol was not simply due to the broad disruption of cell function by an anticancer drug, but that it was related to an effect on Nrf2, because CDDP and MMC did not show an anti-HCV effect as long as they were used at their GI50 concentration. This possibility is also supported by the previous study that demonstrated that the depletion of Nrf2 induced by brusatol was specific and was not a consequence of a broader effect on protein synthesis [32].

To clarify the specificity of brusatol towards Nrf2, the transcriptome of the HPI cells treated with brusatol was compared with that of cells treated with siRNA against Nrf2. In this comparison, 97 genes were commonly down-regulated (less than 0.5-fold vs. control), accounting for 21 and 12% of the whole genes down-regulated by brusatol and the siRNA against Nrf2, respectively. We regarded that these percentages are considerable and significant since 33 of the 97 commonly down-regulated genes were related to cell metabolism especially to lipid metabolism, which is crucial for HCV replication and infection [14, 41, 42]. Moreover, according to the result from the present experiment using the HCV replicon-replicating OR6 cells, inhibition of HCV infection by brusatol is mediated at least via inhibition of HCV replication, which occurs around host lipid droplets [41]. We therefore considered that the reduction of persistent infection of HCV by brusatol was Nrf2-dependent and caused by alteration of host metabolism, especially lipid metabolism, rather than being a nonspecific shutdown by an anticancer drug. However, there still remains a possibility that the gene(s) responsible for the reduction of persistent infection of HCV by brusatol is(are) Nrf2-independent, because the range of gene expression that was affected by brusatol was wider than that affected by knockdown with siRNA against Nrf2.

On the other hand, the transcriptome analysis showed that the number of genes commonly up-regulated by both treatments was unexpectedly large, despite of the fact that brusatol is substantially a negative regulator of transcription. We speculate that the upregulation of these genes by brusatol might be secondary effects of the primary downregulation of the genes affected by brusatol. Moreover, the suppression of HCV infection by brusatol seems to be contradictory to the general concept that Nrf2 promotes host defense against various pathogens. Thus, further study will be needed to identify the gene responsible for brusatol inhibition of HCV persistence and to clarify the precise mechanism by which brusatol inhibits HCV infection.

The present study showed that the inhibition of cell proliferation by brusatol per se was not as potent as that of the other anticancer drugs including MMC, CDDP and sorafenib. This result is possibly due to the difference in the mechanisms of action of these drugs for cell growth inhibition, i.e., static effect by brusatol versus apoptosis by the other anticancer drugs. It has been reported that brusatol enhances the anticancer effect of other anticancer drugs and ameliorates chemoresistance [31, 43, 44]. Consistent with these findings, it was confirmed that brusatol enhanced the inhibition of cell proliferation by other anticancer drugs, indicating that brusatol should be used in combination with an anticancer drug rather than as monotherapy for clinical application. For combination therapy, sorafenib could be the most feasible drug to use together with brusatol, not only because it has been shown to suppress HCV replication, at least in vitro [6–8], as confirmed in the present study but also because sorafenib has already been approved and is used clinically as an anti-HCC drug for systemic therapy [10, 11]. As to the anti-HCV effect, the mechanism by which sorafenib inhibits HCV was shown to be through multistep inhibition throughout the infection cycle of HCV [45]. Clinically, however, sorafenib did not decrease the HCV RNA level in C-HCC patients [13]. A possible explanation for this finding might be that the dose of sorafenib was not sufficient for HCV suppression. There is a clinical possibility that brusatol could augment the effect of HCV reduction by sorafenib.

The present study demonstrated augmentation of the dual anti-HCV and anticancer effects of sorafenib by combination with brusatol, at least in vitro. Their combination achieved almost complete suppression of HCV infection at 72 h after their administration. For clinical application, combination therapy using drugs with a different pharmaceutical mechanism leads to the reduction of adverse effects. We therefore think that brusatol could be clinically applicable in combination with sorafenib for the treatment of HCC, especially when concomitant with HCV infection. However, there are concerns that inhibition of Nrf2 may result in off-target effects on non-cancer cells, thereby causing unexpected adverse effects. Such adverse events may occur not only because Nrf2 is a transcriptional regulator that controls an array of genes including genes involved in host defense and metabolism, but also because brusatol affects a wider range of genes than siRNA against Nrf2. Therefore, for clinical application of brusatol, it will be necessary to extensively clarify its toxicity preclinically in in vitro and in vivo studies.

## Conclusions

This study demonstrated for the first time that the Nrf2 inhibitor brusatol had dual anti-HCV and anticancer effects in vitro and that it enhanced the comparable effects of sorafenib. There is therefore the potential for combination therapy of brusatol with sorafenib for HCC with HCV, in which enhancement of these dual effects by both reagents would be expected. Since brusatol may result in unexpected adverse effects, further studies are required prior to clinical application of brusatol, including studies of its efficacy and safety.

## Additional files

Additional file 1: ** Table S1.** Qunatification of the protein expression in the HPI cell. (XLSX 34 kb)

Additional file 2: ** Figure S1.** Effect of brusatol on the proliferation of HPI cells. Cell viability after the administration of brusatol was determined with the trypan blue staining method. The cell growth rate is presented as fold change relative to that of 0 h. The concentration of GI50 was calculated using the data at 48 h. (PDF 41 kb)

Additional file 3: **Table S2.** Qunatification of the protein expression in the OR6 cell. (XLSX 34 kb)

## Abbreviations

5’UTR: 5′ untranslated region
ATRA: All-trans retinoic acid
CDDP: *Cis*-diamminedichloro-platinum
C-HCC: HCV-positive HCC
DAAs: Direct-acting antivirals
GI50: 50% growth inhibition
HCC: Hepatocellular carcinoma
HCV: Hepatitis C virus
Keap1: Kelch-like ECH-associated protein 1
MMC: Mitomycin C
Nrf2: Nuclear factor E2-related factor 2
NS: Nonstructural
p-Nrf2: Phosphorylated Nrf2
qRT-PCR: Quantitative reverse transcription polymerase chain reaction

## References

[1] El-Serag HB (2012). Epidemiology of viral hepatitis and hepatocellular carcinoma. Gastroenterology.

[2] Ge PS, Runyon BA (2016). Treatment of patients with cirrhosis. N Engl J Med.

[3] Li G, De Clercq E. Current therapy for chronic hepatitis C: the role of direct-acting antivirals. Antivir Res. 2017;142:83–122. 10.1016/j.antiviral.2017.02.014 PMC7172984 28238877

[4] Morgan RL, Baack B, Smith BD, Yartel A, Pitasi M, Falck-Ytter Y (2013). Eradication of hepatitis C virus infection and the development of hepatocellular carcinoma: a meta-analysis of observational studies. Ann Intern Med.

[5] Panel AIHG (2015). Hepatitis C guidance: AASLD-IDSA recommendations for testing, managing, and treating adults infected with hepatitis C virus. Hepatology.

[6] Wilhelm S, Carter C, Lynch M, Lowinger T, Dumas J, Smith RA, Schwartz B, Simantov R, Kelley S (2006). Discovery and development of sorafenib: a multikinase inhibitor for treating cancer. Nat Rev Drug Discov.

[7] Himmelsbach K, Hildt E (2013). The kinase inhibitor Sorafenib impairs the antiviral effect of interferon alpha on hepatitis C virus replication. Eur J Cell Biol.

[8] Himmelsbach K, Sauter D, Baumert TF, Ludwig L, Blum HE, Hildt E (2009). New aspects of an anti-tumour drug: sorafenib efficiently inhibits HCV replication. Gut.

[9] Burckstummer T, Kriegs M, Lupberger J, Pauli EK, Schmittel S, Hildt E (2006). Raf-1 kinase associates with hepatitis C virus NS5A and regulates viral replication. FEBS Lett.

[10] Llovet JM, Ricci S, Mazzaferro V, Hilgard P, Gane E, Blanc JF, de Oliveira AC, Santoro A, Raoul JL, Forner A (2008). Sorafenib in advanced hepatocellular carcinoma. N Engl J Med.

[11] Cheng AL, Kang YK, Chen Z, Tsao CJ, Qin S, Kim JS, Luo R, Feng J, Ye S, Yang TS (2009). Efficacy and safety of sorafenib in patients in the Asia-Pacific region with advanced hepatocellular carcinoma: a phase III randomised, double-blind, placebo-controlled trial. Lancet Oncol.

[12] Hollebecque A, Cattan S, Romano O, Sergent G, Mourad A, Louvet A, Dharancy S, Boleslawski E, Truant S, Pruvot FR (2011). Safety and efficacy of sorafenib in hepatocellular carcinoma: the impact of the child-Pugh score. Aliment Pharmacol Ther.

[13] Cabrera R, Limaye AR, Horne P, Mills R, Soldevila-Pico C, Clark V, Morelli G, Firpi R, Nelson DR (2013). The anti-viral effect of sorafenib in hepatitis C-related hepatocellular carcinoma. Aliment Pharmacol Ther.

[14] Sugiyama K, Ebinuma H, Nakamoto N, Sakasegawa N, Murakami Y, Chu PS, Usui S, Ishibashi Y, Wakayama Y, Taniki N (2014). Prominent steatosis with hypermetabolism of the cell line permissive for years of infection with hepatitis C virus. PLoS One.

[15] Itoh K, Chiba T, Takahashi S, Ishii T, Igarashi K, Katoh Y, Oyake T, Hayashi N, Satoh K, Hatayama I (1997). An Nrf2/small Maf heterodimer mediates the induction of phase II detoxifying enzyme genes through antioxidant response elements. Biochem Biophys Res Commun.

[16] Uruno A, Motohashi H (2011). The Keap1-Nrf2 system as an in vivo sensor for electrophiles. Nitric Oxide.

[17] Mitsuishi Y, Taguchi K, Kawatani Y, Shibata T, Nukiwa T, Aburatani H, Yamamoto M, Motohashi H (2012). Nrf2 redirects glucose and glutamine into anabolic pathways in metabolic reprogramming. Cancer Cell.

[18] Kobayashi A, Kang MI, Watai Y, Tong KI, Shibata T, Uchida K, Yamamoto M (2006). Oxidative and electrophilic stresses activate Nrf2 through inhibition of ubiquitination activity of Keap1. Mol Cell Biol.

[19] Martin D, Rojo AI, Salinas M, Diaz R, Gallardo G, Alam J, De Galarreta CM, Cuadrado A (2004). Regulation of heme oxygenase-1 expression through the phosphatidylinositol 3-kinase/Akt pathway and the Nrf2 transcription factor in response to the antioxidant phytochemical carnosol. J Biol Chem.

[20] Zhou L, Yang Y, Tian D, Wang Y (2013). Oxidative stress-induced 1, N6-ethenodeoxyadenosine adduct formation contributes to hepatocarcinogenesis. Oncol Rep.

[21] Jaramillo MC, Zhang DD (2013). The emerging role of the Nrf2-Keap1 signaling pathway in cancer. Genes Dev.

[22] Wang J, Zhang M, Zhang L, Cai H, Zhou S, Zhang J, Wang Y (2010). Correlation of Nrf2, HO-1, and MRP3 in gallbladder cancer and their relationships to clinicopathologic features and survival. J Surg Res.

[23] Zhang M, Zhang C, Zhang L, Yang Q, Zhou S, Wen Q, Wang J (2015). Nrf2 is a potential prognostic marker and promotes proliferation and invasion in human hepatocellular carcinoma. BMC Cancer.

[24] Chen J, Yu Y, Ji T, Ma R, Chen M, Li G, Li F, Ding Q, Kang Q, Huang D (2016). Clinical implication of Keap1 and phosphorylated Nrf2 expression in hepatocellular carcinoma. Cancer Med.

[25] Singh A, Misra V, Thimmulappa RK, Lee H, Ames S, Hoque MO, Herman JG, Baylin SB, Sidransky D, Gabrielson E (2006). Dysfunctional KEAP1-NRF2 interaction in non-small-cell lung cancer. PLoS Med.

[26] Kim YR, Oh JE, Kim MS, Kang MR, Park SW, Han JY, Eom HS, Yoo NJ, Lee SH (2010). Oncogenic NRF2 mutations in squamous cell carcinomas of oesophagus and skin. J Pathol.

[27] Totoki Y, Tatsuno K, Covington KR, Ueda H, Creighton CJ, Kato M, Tsuji S, Donehower LA, Slagle BL, Nakamura H (2014). Trans-ancestry mutational landscape of hepatocellular carcinoma genomes. Nat Genet.

[28] Schulze K, Imbeaud S, Letouze E, Alexandrov LB, Calderaro J, Rebouissou S, Couchy G, Meiller C, Shinde J, Soysouvanh F (2015). Exome sequencing of hepatocellular carcinomas identifies new mutational signatures and potential therapeutic targets. Nat Genet.

[29] Ivanov AV, Bartosch B, Smirnova OA, Isaguliants MG, Kochetkov SN (2013). HCV and oxidative stress in the liver. Viruses.

[30] Saito T, Ichimura Y, Taguchi K, Suzuki T, Mizushima T, Takagi K, Hirose Y, Nagahashi M, Iso T, Fukutomi T (2016). p62/Sqstm1 promotes malignancy of HCV-positive hepatocellular carcinoma through Nrf2-dependent metabolic reprogramming. Nat Commun.

[31] Ren D, Villeneuve NF, Jiang T, Wu T, Lau A, Toppin HA, Zhang DD (2011). Brusatol enhances the efficacy of chemotherapy by inhibiting the Nrf2-mediated defense mechanism. Proc Natl Acad Sci U S A.

[32] Olayanju A, Copple IM, Bryan HK, Edge GT, Sison RL, Wong MW, Lai Z-Q, Lin Z-X, Dunn K, Sanderson CM (2015). Brusatol provokes a rapid and transient inhibition of Nrf2 signaling and sensitizes mammalian cells to chemical toxicity—implications for therapeutic targeting of Nrf2. Free Radic Biol Med.

[33] Ikeda M, Abe K, Dansako H, Nakamura T, Naka K, Kato N (2005). Efficient replication of a full-length hepatitis C virus genome, strain O, in cell culture, and development of a luciferase reporter system. Biochem Biophys Res Commun.

[34] Shima N, Stolz DB, Miyazaki M, Gohda E, Higashio K, Michalopoulos GK (1998). Possible involvement of p21/waf1 in the growth inhibition of HepG2 cells induced by hepatocyte growth factor. J Cell Physiol.

[35] Blight KJ, McKeating JA, Rice CM (2002). Highly permissive cell lines for subgenomic and genomic hepatitis C virus RNA replication. J Virol.

[36] Wang XJ, Hayes JD, Henderson CJ, Wolf CR (2007). Identification of retinoic acid as an inhibitor of transcription factor Nrf2 through activation of retinoic acid receptor alpha. Proc Natl Acad Sci U S A.

[37] Tang X, Wang H, Fan L, Wu X, Xin A, Ren H, Wang XJ (2011). Luteolin inhibits Nrf2 leading to negative regulation of the Nrf2/ARE pathway and sensitization of human lung carcinoma A549 cells to therapeutic drugs. Free Radic Biol Med.

[38] Arlt A, Sebens S, Krebs S, Geismann C, Grossmann M, Kruse ML, Schreiber S, Schafer H (2013). Inhibition of the Nrf2 transcription factor by the alkaloid trigonelline renders pancreatic cancer cells more susceptible to apoptosis through decreased proteasomal gene expression and proteasome activity. Oncogene.

[39] Li M, Sun Y, Guan X, Shu X, Li C (2014). Advanced progress on the relationship between RA and its receptors and malignant tumors. Crit Rev Oncol Hematol.

[40] Willingham W, Considine RT, Chaney SG, Lee KH, Hall IH (1984). Reversibility of protein synthesis inhibition by quassinoid antineoplastic agents in a rabbit reticulocyte system. Biochem Pharmacol.

[41] Ogawa K, Hishiki T, Shimizu Y, Funami K, Sugiyama K, Miyanari Y, Shimotohno K (2009). Hepatitis C virus utilizes lipid droplet for production of infectious virus. Proc Jpn Acad Ser B Phys Biol Sci.

[42] Shimizu Y, Hishiki T, Ujino S, Sugiyama K, Funami K, Shimotohno K (2011). Lipoprotein component associated with hepatitis C virus is essential for virus infectivity. Curr Opin Virol.

[43] Wu T, Harder BG, Wong PK, Lang JE, Zhang DD (2015). Oxidative stress, mammospheres and Nrf2–new implication for breast cancer therapy?. Mol Carcinog.

[44] Tao S, Wang S, Moghaddam SJ, Ooi A, Chapman E, Wong PK, Zhang DD (2014). Oncogenic KRAS confers chemoresistance by upregulating NRF2. Cancer Res.

[45] Descamps V, Helle F, Louandre C, Martin E, Brochot E, Izquierdo L, Fournier C, Hoffmann TW, Castelain S, Duverlie G (2015). The kinase-inhibitor sorafenib inhibits multiple steps of the hepatitis C virus infectious cycle in vitro. Antivir Res.

