# Supplementary figures and images for: Dual effects of the Nrf2 inhibitor for inhibition of hepatitis C virus and hepatic cancer cells
Source: BMC Cancer. 2018 Jun 25;18:680. doi: 10.1186/s12885-018-4588-y (PMC6019801; doi:10.1186/s12885-018-4588-y)

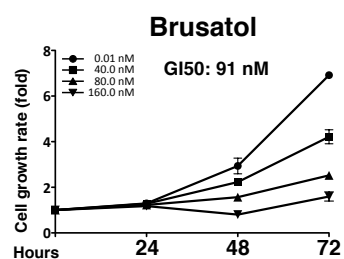

Supplement: Supplementary file 2 — Figure S1. Effect of brusatol on the proliferation of HPI cells. Cell viability after the administration of brusatol was determined with the trypan blue staining method. The cell growth rate is presented as fold change relative to that of 0 h. The concentration of GI50 was calculated using the data at 48 h. (PDF 41 kb) [file 12885_2018_4588_MOESM2_ESM.pdf]
